# Chitinase (CHI) of *Spodoptera frugiperda* affects molting development by regulating the metabolism of chitin and trehalose

**DOI:** 10.3389/fphys.2022.1034926

**Published:** 2022-10-03

**Authors:** Xiang-Yu Liu, Sha-Sha Wang, Fan Zhong, Min Zhou, Xin-Yi Jiang, Yi-Sha Cheng, Yi-Hao Dan, Gao Hu, Can Li, Bin Tang, Yan Wu

**Affiliations:** ^1^ Guizhou Provincial Key Laboratory for Rare Animal and Economic Insect of the Mountainous Region, Department of Biology and Engineering of Environment, Guiyang University, Guiyang, China; ^2^ College of Life and Environmental Sciences, Hangzhou Normal University, Hangzhou, Zhejiang, China; ^3^ College of Plant Protection, Nanjing Agricultural University, Nanjing, China

**Keywords:** RNAi, chitinase, trehalase, chitin synthesis, qRT-PCR, *Spodoptera frugiperda*

## Abstract

Chitin is the main component of insect exoskeleton and midgut peritrophic membrane. Insect molting is the result of the balance and coordination of chitin synthesis and degradation in chitin metabolism under the action of hormones. In this study, a 678 bp dsRNA fragment was designed and synthesized according to the known CHI (Chitinase) sequence of *Spodoptera frugiperda*. It was injected into the larvae to observe the molting and development of *S. frugiperda*. At the same time, the activities of trehalase and chitinase, the contents of trehalose, chitin and other substances were detected, and the expression of related genes in the chitin synthesis pathway was determined. The results showed that *CHI* gene was highly expressed at the end of each instar, prepupa and pupal stage before molting; At 12 and 24 h after dsRNA injection of *CHI* gene of *S. frugiperda*, the expression of *CHI* gene decreased significantly, and the chitinase activity decreased significantly from 12 to 48 h. The expression of chitin synthase (*CHSB*) gene decreased significantly, and the chitin content increased significantly. Some larvae could not molt normally and complete development, leading to certain mortality. Secondly, after RNAi of *CHI* gene, the content of glucose and glycogen increased first and then decreased, while the content of trehalose decreased significantly or showed a downward trend. The activities of the two types of trehalase and the expression levels of trehalase genes decreased first and then increased, especially the trehalase activities increased significantly at 48 h after dsCHI injection. And trehalose-6-phosphate synthase (*TPS*), glutamine: fructose-6-phosphate amidotransferase (*GFAT*), UDP-N-acetylglucosamine pyrophosphorylases (*UAP*), hexokinase (*HK*), glucose-6-phosphate isomerase (*G6PI*) and phosphoacetylglucosamine mutase (*PAGM*) all decreased significantly at 24 h, and then increased or significantly increased at 48 h. These results indicated that when the expression of chitinase gene of *S. frugiperda* was inhibited, it affected the degradation of chitin in the old epidermis and the formation of new epidermis, and the content of chitin increased, which led to the failure of larvae to molt normally. Moreover, the chitin synthesis pathway and trehalose metabolism were also regulated. The relevant results provide a theoretical basis for screening target genes and developing green insecticides to control pests by using the chitin metabolism pathway.

## Introduction

Chitin is the most widely distributed amino polysaccharide in nature and the second largest polysaccharide after cellulose (Lee, 2009). In addition to plants and higher animals, it exists in a variety of organisms. It is a polycrystalline substance containing α-, β- and γ three forms. In insects, chitin is mainly used as a scaffold material to interact with other protein mechanisms and form an important part of insect epidermis, trachea and peritrophic membrane (Zhu et al., 2016). Its main functions include: first, it plays an important role in the growth and development of insects, especially in the process of periodic molting (Arakane and Muthukrishanan, 2010); Second, as the main component of the body surface or trachea epidermis, it protects the internal organs of insects and prevents the invasion of foreign toxins and pathogens to organisms (Hegedus et al., 2009; Doucet and Retnakaran, 2012). Based on the important role of chitin in insect growth and development, and the absence of chitin in higher animals, the control of chitin synthesis and degradation has become a safe target for the development of new pesticides (Oyeleye and Normi, 2018; Zhang X. et al., 2021; Zhang J. Y. et al., 2021; Wang et al., 2022). In addition, the purified chitinase has certain insecticidal activity (Suganthi et al., 2017), which has attracted more and more attention.

The exoskeleton of insects is mainly composed of chitin. The regular molting is to take off the old exoskeleton and form a new exoskeleton. This process is completed through the synergistic action of various enzymes involved in chitin synthesis and degradation (Li et al., 2022). Chitin biosynthesis in insects starts with trehalose as precursor and trehalase (Tang et al., 2012). After a series of physiological and biochemical reactions, chitin is finally synthesized by chitin synthase (CHS) (Zhang W. Q. et al., 2011; Zhu et al., 2016). Chitinase (EC. 3.2.1.14) (CHI/Cht) is a kind of glycosyl hydrolase that can hydrolyze chitin. Its size is between 20 kd and 90 kd, and it exists in various organisms, including bacteria, fungi, yeast, plants, arthropods and humans (Merzendorfer, 2013). According to the different cleavage sites of chitinase, it can be divided into endo chitinase and exo chitinase (Chen et al., 2018), which simultaneously function to degrade chitin (Mi et al., 2015). At the same time, according to the homology of amino acids in the catalytic region of chitinases in various organisms, chitinases are generally divided into family 18 and family 19 glycosylhydrolases (Henrissat and Bairoch, 1993). However, most of the highly efficient chitin degradation systems in insects belong to family 18 chitinases (Kramer and Muthukfishnan, 1997). The glycoside hydrolase family 48 were isolated from the active adults of the leaf beetle *Gastrophysa atrocyanea* (Fujita et al., 2006). Common chitinases and chitinase-like proteins are mostly endo chitinases and contain some conserved domains, catalytic domains, cysteine-rich chitin-binding domains, and serine/threonine-rich linker regions (Arakane and Muthukrishanan, 2010). According to phylogenetic analysis and protein structure characteristics, it is mainly divided into eight groups, group I-Ⅷ. The chitinase functions of different groups are different (Merzendorfer, 2013; Li et al., 2022). Under the control of ecdysone and juvenile hormone, endochitinase acts on β-(1,4) glycosidic linkages, hydrolyzing chitin to chitosan, and then hydrolyzed by exonuclease β-N-acetylglucosamine monomer, and then chitin synthase acts to synthesize new epidermis (Dahiya et al., 2006; Arakane and Muthukrishanan, 2010), to ensure normal molting and development of insects.

The most basic function of chitinase in insects is to change the chitin structure, participate in innate immunity and development (Lee, 2009; Adrangi and Faramarzi, 2013), especially play a very important role in the process of molting, and participate in the replacement of new and old epidermis and the formation of peritrophic membrane (Zhu et al., 2008a). Insect chitinase is not only expressed in various organs or tissues, including molting gland and midgut (Zhang et al., 2011b), but also expressed intermittently throughout the development cycle, especially in the early molting stage (Bolognesi et al., 2005). Chitinase expressed in the body wall is mainly related to insect molting and embryo development and hatching of eggs (Zhu et al., 2008b; Zhang et al., 2018). Chitinases in the midgut are mainly responsible for chitin degradation in the peritrophic membrane and intestinal wall during development (Ren et al., 2014). In the molting stage of insects, several functional specific chitinases are highly expressed and participate in the degradation of the old epidermis. The actual synthesis of the new epidermis and the degradation of the old epidermis occur simultaneously. It is very important to protect the new epidermis from degradation by chitinases, mainly through the direct interaction of specific proteins with the newly synthesized chitin to prevent the degradation of the external chitinases (Petkau et al., 2012; Mi et al., 2015). Therefore, chitinase usually plays a role in the early stage of molting. If chitinase is continuously expressed and acts, the synthesis of chitin may still be affected after the formation of new epidermis, thus causing the development of insects to be blocked and even death (Xi et al., 2015; Liu et al., 2017). Many studies have found that once the expression of chitinase gene was inhibited, the development of insect larvae such as molting, pupation, adult eclosion and wings would be affected (Zhu et al., 2008b; Pesch et al., 2016; Dong et al., 2020; Zhang X. et al., 2021).

*Spodoptera frugiperda* (J.E. Smith), as a major migratory pest newly invading China, has expanded the area of damage and rapidly become a frequent important pest to corn (Wu et al., 2021; Guo et al., 2022). RNAi technology has been more and more widely used in the study of insect gene function (Zhang et al., 2021). Recently, with the occurrence of the harm of *S. frugiperda*, more and more attention has been paid to screening and studying the target genes of control by using RNAi technology (Ghosh et al., 2016; Dhandapani et al., 2022). In addition, the chitin synthesis and degradation pathway of insects has always been a hot and important field in the research of pest control targets. No matter how different the structure and activity of each chitinase are (Zhu et al., 2008a), as long as it can degrade chitin, it can affect the chitin metabolism of insects and affect the molting and development differently (Xi et al., 2015), but all of them jointly regulate the balance of insect chitin (Zhang et al., 2016). When the function of *CHI* gene of *S. frugiperda* was studied by the bacterial-mediated RNAi technology previously reported, it was found that the inhibition of *CHI* gene expression could affect adult eclosion and molt (Wan et al., 2021). In this study, it is planned to further use RNAi technology to inhibit the expression of *CHI* gene in the larvae of *S. frugiperda*, and explore whether it regulates the molting development of the larvae, so as to lay a foundation for screening target genes and developing green insecticides to prevent and control *S. frugiperda* in the future.

## Materials and methods

### Test insects

The source of *S. frugiperda* was from Zhejiang Academy of Agricultural Sciences. The larvae and adults were raised in the light incubator. Feeding conditions: temperature 25 ± 1°C, relative humidity 60 ± 10%, photoperiod 16L: 8D. Larvae were fed artificial feed (Pinto et al., 2019; Wang et al., 2022), and adults were fed 10% honey water.

### RNA extraction and cDNA synthesis

Total RNA in *S. frugiperda* was extracted according to the instructions of RNAiso Plus (Invitrogen, Carlsbad, CA, United States) reagent. Subsequently, the quality of the total RNA was detected with 1% agarose, and the concentration and purity of the extracted RNA were measured with a micro nucleic acid analyzer NanoDrop^™^ 2000. Taking 1.0 μg total RNA was used as a template, and the first strand of cDNA was reverse transcribed and synthesized according to the instructions of PrimeScript^™^ RT Reagent Kit with gDNA Eraser (Takara, Kyoto, Japan) kit.

### Synthesis of dsRNA and microinjection

Double stranded RNA (dsRNA) of *CHI* (GenBank ID: XM_035597059.1) specific primers were designed for PCR reactions using the primer three web online website (Supplementary Table S1). The size of dsCHI is 678 bp. After agarose gel electrophoresis, the specific and appropriate fragment size bands were cut and recovered according to the MiniBEST Agarose Gel DNA Extraction Kit Ver. 4.0 (Takara, Kyoto, Japan). The recovered PCR products were ligated and transformed using the vector pMD^®^18-T and DH5α competent cells. Then, the bacterial solutions verified by PCR were sent to Zhejiang Shangya Biotechnology Co., Ltd. for sequencing and returned to the plasmids. The primers with T7 promoter were used to perform cross PCR reaction on the correctly sequenced plasmid, and then the dsRNA of the target gene was synthesized according to the instructions of the T7 RiboMAX^™^ Express RNAi System (Promega, Madison, WI, United States). Meanwhile, a 657 bp dsGFP (GenBank ID: MN728541.1) was synthesized by the same method with dsRNA injected with green fluorescent protein (GFP) gene as the control group.

**FIGURE 1:**
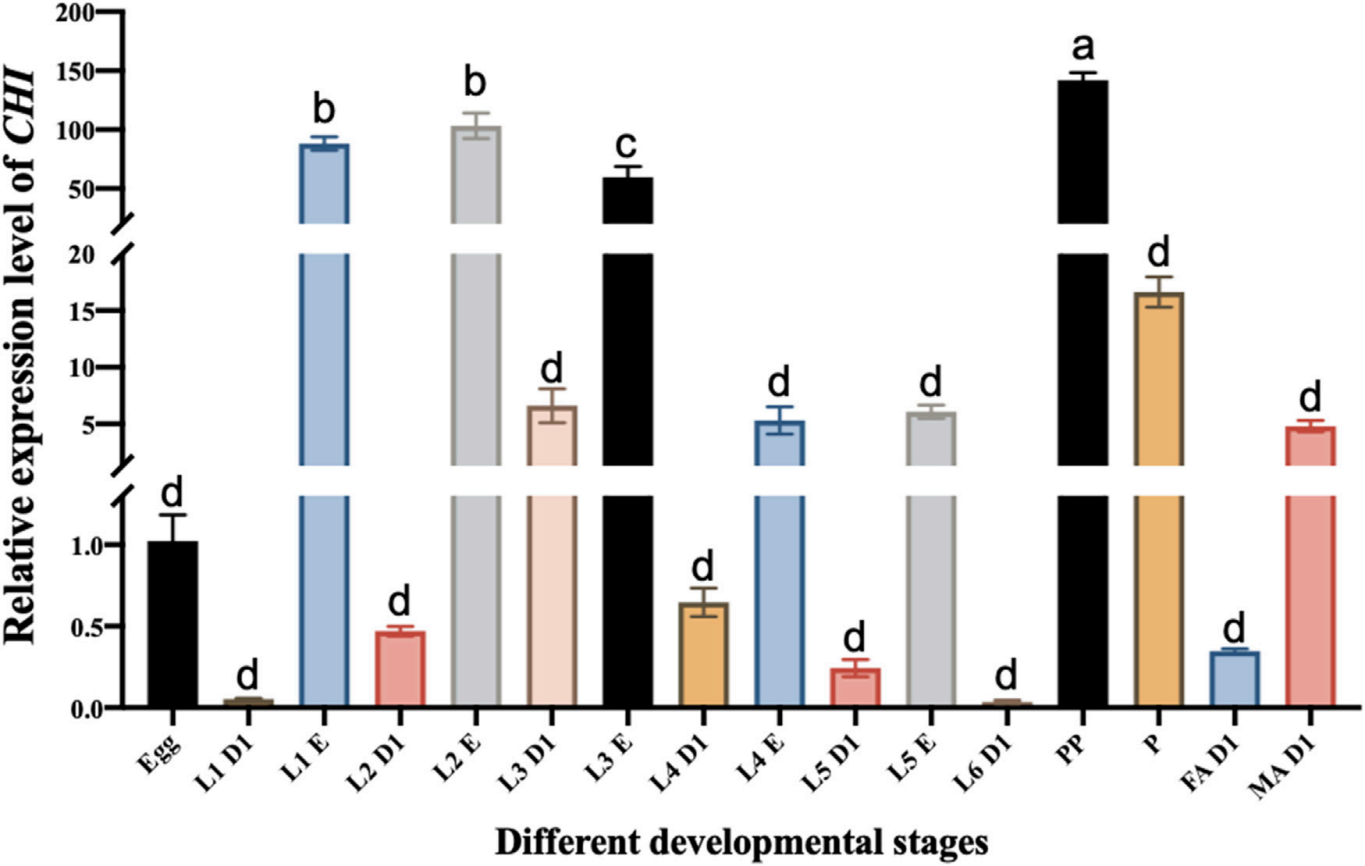
Changes in the expression level of *CHI* in different developmental stages of *Spodoptera frugiperda.* L1 D1, Larvae at the first day of the first instar; L1 E, Larvae at the end of the first instar; L2 D1, Larvae at the first day of the second instar; L2 E, Larvae at the end of the second instar; L3 D1, Larvae at the first day of the third instar; L3 E, Larvae at the end of the third instar; L4 D1, Larvae at the first day of the fourth instar; L4 E, Larvae at the end of the fourth instar; L5 D1, Larvae at the first day of the fifth instar; L5 E, Larvae at the end of the fifth instar; L6 D1, Larvae at the first day of the sixth instar; PP, prepupae; P, pupae; FA D1, Female adults at the first day; MA D1, Male adults at the first day. Three replicates were performed per group. (Mean ± SE; Tukey’s test; Different letters in the figure indicated significant differences between groups, *p* < 0.05).

Microinjection was performed using Eppendorf TransferMan ^®^ 4r microinjection system. According to previous reports, the inhibition of *CHI* gene expression can affect the emergence of the adults of *S. frugiperda* (Wan et al., 2021). The purpose of this experiment is to explore the influence of *CHI* on larvae after RNAi, authors chose the third instar larvae that are suitable for injection as the experimental object, and the expression level of *CHI* in the third instar larvae was higher than that of the fourth, fifth and sixth instar larvae (Figure 1). The injection site was between the second and third pairs of thorax feet. The injection volume was 300 nl (the concentration was about 2500 ng·μL-1). About 30 individuals were injected each time. Samples were taken at 12, 24 and 48 h after injection for subsequent experiments.

### Collection of experimental materials and phenotypic observation

Eggs, larvae on the first day of first-sixth instar (40, 10, 5, 3, 3 and 3 individuals per sample respectively), larvae at the end of first-fifth instar (10, 5, 3, 3 and 3 individuals per sample respectively), prepupa (three individuals per sample), pupa (three individuals per sample), female adults on the first day and male adults on the first day (three individuals per sample) were collected to determine the expression pattern of *CHI* during the development of *S. frugiperda*. Three biological replicates were performed. After injection of dsRNA, the development of larvae was continuously observed and the survival rate was counted. In addition, the abnormal phenotypes were photographed using Leica EZ4 HD stereomicroscope and LAS EZ software.

### qRT-PCR

Gene expression at the mRNA level was detected with TB Green^®^ Premix Ex Taq^™^ (Tli RNaseH Plus) kit (Takara, Kyoto, Japan) in a Bio-Rad CFX96 Real-Time PCR Detection System (Bio-Rad Laboratories Inc., Hercules, CA, United States). qRT-PCR system: TB Green 10 μl, cDNA of *S. frugiperda* 1 μl, forward primer 1 μl, reverse primer 1 μl, Sterilized wate 7 μl qRT-PCR procedure: preincubation at 95°C for 30 s; 40 cycles of 95°C for 5 s and annealing at 50–60°C for 30 s; and a melting curve at 65–95°C. Ribosomal protein L10 (RPL10, GenBank ID: OK319023.1) was used as the internal reference gene (Gurusamy et al., 2020). The gene primer sequence is shown in Supplementary Table S1. Three biological replicates and three technical replicates were performed. qRT-PCR data were obtained using 2^−ΔΔCT^ method (Livak and Schmittgen, 2001).

### Determination of carbohydrate content and trehalase activity

Four-five individuals after injection were put into the same centrifuge tube, and three biological replicates were performed. For each sample, 200 μl of phosphate-buffered saline (PBS) was added. The mixture was thoroughly ground with a grinding rod and placed in a sonicator for sonication, and then supplemented to 1 ml with PBS. At 4°C, 1000 × *g* for 20 min, and the supernatant was transferred to two centrifuge tubes, 350 μl each. One part of the supernatant was used to detect trehalose, glycogen and protein content, and the other part of the supernatant was at 4°C 20800 × *g* for 60 min. Then take 300 μl supernatant was used to detect glucose, protein content and soluble trehalase activity, and 300 μl PBS was mixed to prepare suspension, which was used to detect glucose, protein content and membrane-bound trehalase activity. Trehalose content was detected by anthrone method, glucose, glycogen and enzyme activity were detected by Glucose Assay Kit (Sigma, MO, United States), and protein content was detected by BCA Protein Assay Kit (Beyotime, Shanghai, China). The specific experimental steps can be found in the study of Zhang et al. (2017).

### Determination of chitinase activity and chitin content

Four-five individuals after injection were put into the same centrifuge tube, and three biological replicates were performed. The activity of chitinase in *S. frugiperda* was determined according to the instructions of the chitinase kit (Comin, Suzhou, China). In addition, the protein concentration in the sample was detected according to the BCA protein concentration determination kit (Beyotime, Shanghai, China). Finally, chitinase activity was calculated according to protein concentration.

For chitin content determination, refer to Arakane et al. (2005). Four-five individuals after injection were put into the same centrifuge tube, and three biological replicates were performed. Firstly, liquid nitrogen was used to grind and crush the larva of *S. frugiperda*, then 0.5 ml of 6% KOH was added, and the larva was violently shaken for 5 min, and then the larva was bathed in water at 80°C for 90 min. Then centrifuged at 4°C at 12,000 × *g* for 20 min, and the supernatant was poured out. Add 1 ml 1 × PBS to resuspend the precipitate, centrifuge at 4°C at 12,000 × *g* for 20 min, and pour out the supernatant. Add 200 µl McIlvaine’s buffer and 25 µl 5 mg/ml Chitinase from *Streptomyces* Griseus (Sigma, MO, United States) to the precipitate, mix well and react at 37°C for 72 h to hydrolyze chitin into GlcNAc. After the reaction, the samples were centrifuged at 25°C at 12,000 × *g* for 10 min. Meanwhile, standard GlcNAc solutions with concentrations of 0, 20, 40, 60, 80, 100 and 120 µg/ml were prepared. Suck 60 µl of sample supernatant and standard GlcNAc solution into a new 1.5 ml centrifuge tube. Then 60 µl 0.27 M sodium borate was added respectively, and centrifuged at 25°C and 12,000 × *g* for 1 min after mixing. Put the centrifuge tube in the 99.9°C water bath for 1 min, and then mix well, and continue to put it in the 99.9°C water bath for 10 min. After cooling to room temperature, 600 µl of diluted dimethylaminobenzaldehyde (1×DMAB) was added and incubated at 37°C for 20 min. Finally, 200 µl was aspirated to determine the absorbance value at 585 nm.

### Data analysis

Independent sample *t*-test or one-way ANOVA followed by the Tukey’s test in IBM SPSS Statistics 20 was used to analyze the difference significance of the data. Then the results were plotted using the software graphpad prism version 8.4.0. The data in the figure are expressed as mean ± standard error (Mean ± SE), and different letters in the figure indicate significant differences between groups (*p* < 0.05). The analysis results of *t*-test are indicated by *, *** stands for *p* < 0.001, ** stands for *p* < 0.01, * stands for *p* < 0.05, and “ns” stands for *p* > 0.05.

## Results

### Analysis of ***CHI*** expression during the different stages in *S. frugiperda*

The *CHI* expression of different stages of the development of *S. frugiperda* was detected. It was found that the *CHI* expression at the beginning of each instar was significantly lower than that at the end of the instar. For example, there was a significant difference between them at the beginning of the first instar and the end of the first instar (Figure 1). This is consistent with the fact that the larva of *S. frugiperda* needs to shed its old skin at the end of each instar, which just needs high expression of chitinase to play a role. It also indirectly indicates that *CHI* is one of the key degradation genes for the epidermal ecdysis development of *S. frugiperda* larvae. However, the expression level of *CHI* in the whole development stage is the highest in the prepupa stage, and there is also high expression in the pupal stage (Figure 1). This result is similar to that of Bolognesi et al. (2005), *CHI* of *S. frugiperda* was expressed during the wandering and pupal stages. During this period, no ecdysis occurred in the epidermis. It is estimated that the high expression of *CHI* is related to the development of trachea and peritrophic membrane from larvae to adults, and further research is needed.

### Analysis of RNAi effect of ***CHI*** gene in *S. frugiperda*

**FIGURE 2:**
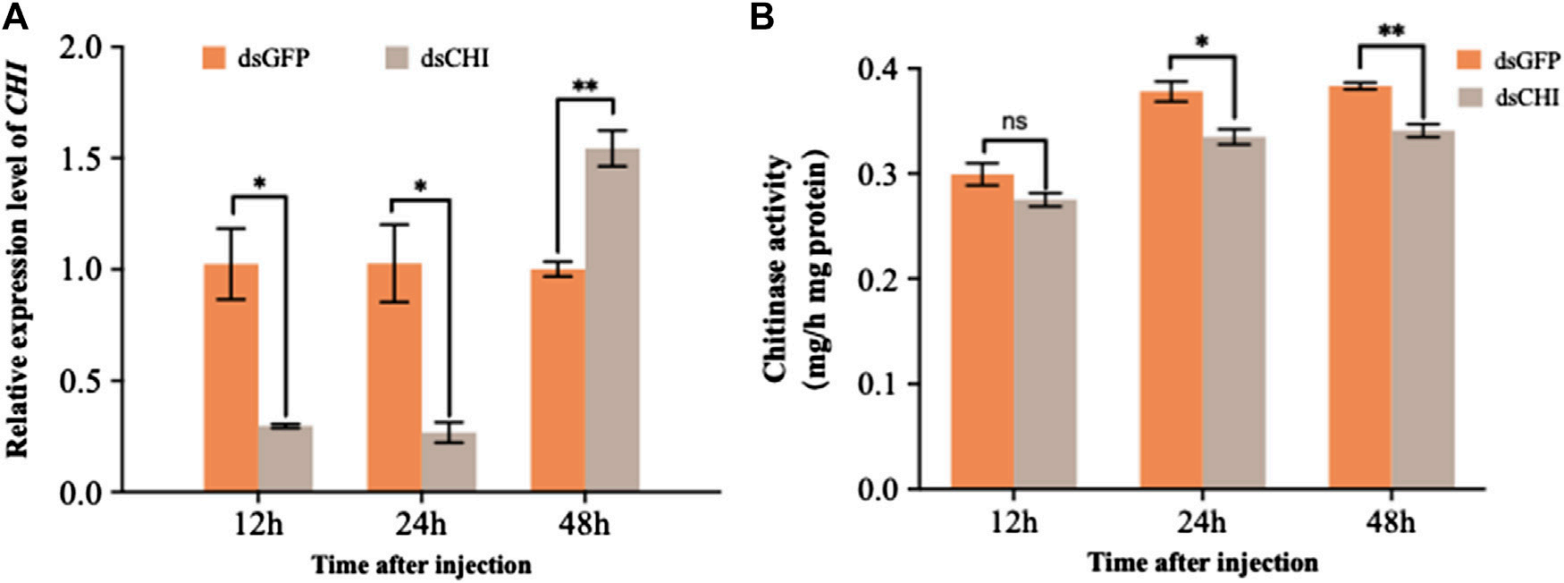
Changes in the expression and activity of chitinase after RNAi. *Spodoptera frugiperda* larvae at the first day of the third instar stage were divided into three groups and injected with dsGFP and dsCHI, respectively. Insects were collected and used to determine the relative expression level of chitinase **(A)** and chitinase activity **(B)** at 12, 24 and 48 h after dsRNA injection. Three replicates were performed per group. (Mean ± SE; t - test; **p < 0.01, *p < 0.05, ns *p* > 0.05).

According to the sequence of *CHI* gene (GenBank ID: XM_035597059.1), a 678 bp fragment was selected to synthesize dsRNA for RNAi experiment of this gene. The results showed that the expression of *CHI* gene decreased significantly at 12 and 24 h after injection of dsRNA of *CHI* gene, and then increased significantly at 48 h compared with the control group (Figure 2A). Further measuring the chitinase activity, it was found that the chitinase activity in *S. frugiperda* decreased significantly and extremely significantly at 24 and 48 h after injection of dsCHI (Figure 2B), indicating that the synthesized dsRNA fragment of *CHI* gene can effectively inhibit the chitinase activity of *S. frugiperda*.

**FIGURE 3:**
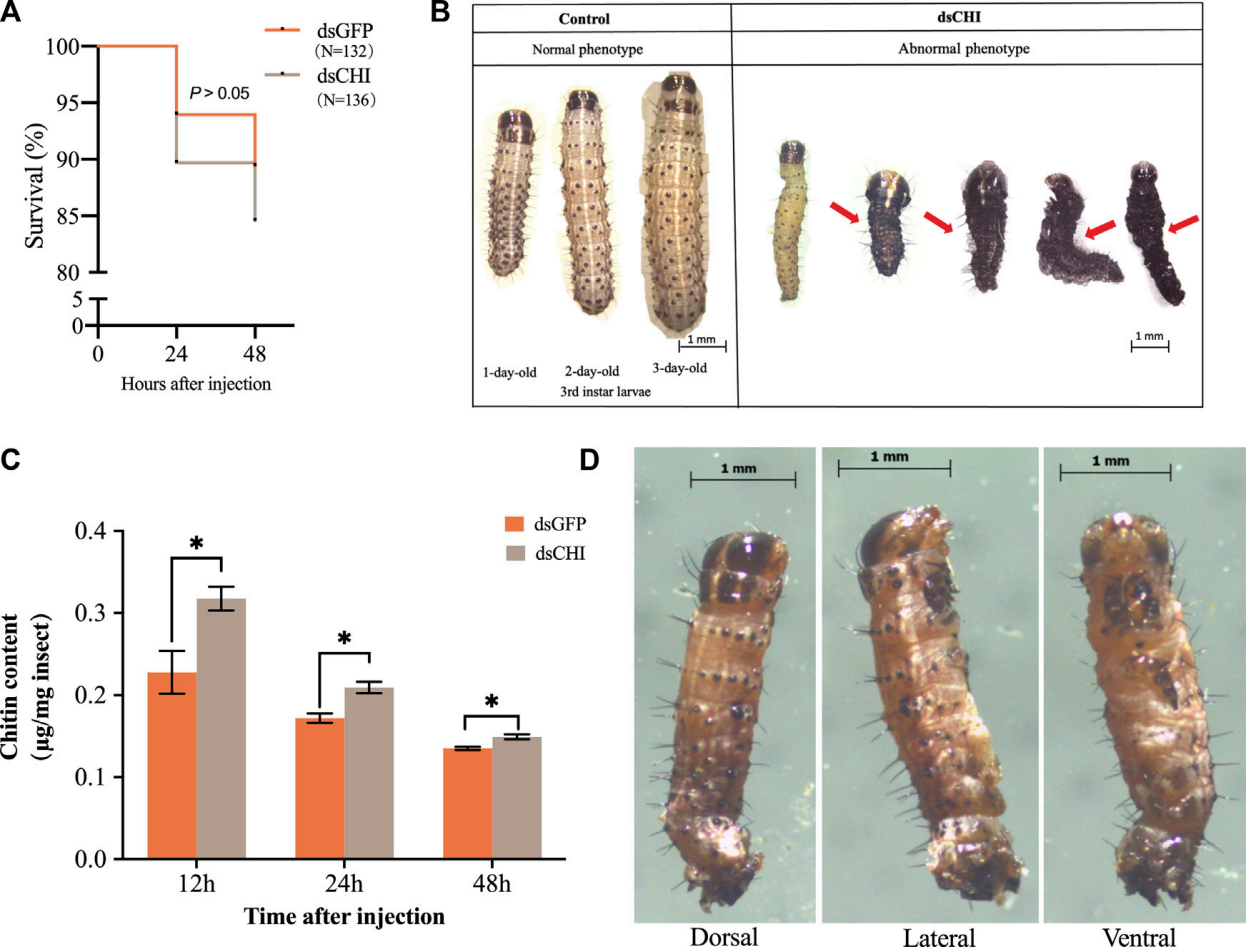
Survival **(A)**, abnormal phenotype **(B**,**D)** and chitin content **(C)** changes of *Spodoptera frugiperda* after RNAi. The numbers of *Spodoptera frugiperda* larvae used to count the survival rate of dsGFP and dsCHI groups were 132 and 136, respectively. Larvae at the first day of the third instar stage were divided into three groups and injected with dsGFP and dsCHI, respectively. Insects were collected and used to determine chitin content **(C)**. Three replicates were performed per group. Figure 3D showed one type of death phenotype in the dsCHI treatment. (Mean ± SE; t-test; **p < 0.01, *p < 0.05, ns *p* > 0.05).

Although not statistically significant (*p* > 0.05), the survival rate of *S. frugiperda* larvae at 48 h was 5% lower than that of the control group after dsRNA injection of *CHI* gene (Figure 3A), indicating that the decrease of *CHI* expression of *S. frugiperda* will lead to certain death, which is actually due to the inhibition of the degradation of epidermis. The larvae had difficulty in molting, and some of them couldn’t complete the molting process and develop (Figure 3B). Further studies showed that after *CHI* expression was reduced, chitin content increased significantly (Figure 3C), and some larvae could molt normally, develop normally, move slowly and die. After RNAi, a death phenotype was observed, that is, the larva was full of yellow liquid and solid mixture, not the tissue fluid in normal development. (Figure 3D), which indicated that *CHI* of *S. frugiperda* played a role in the degradation of chitin in the midgut peritrophic membrane, which was consistent with previous reports (Bolognesi et al., 2005).

### Analysis of carbohydrate content change after RNAi of ***CHI*** gene of *S. frugiperda*

**FIGURE 4:**
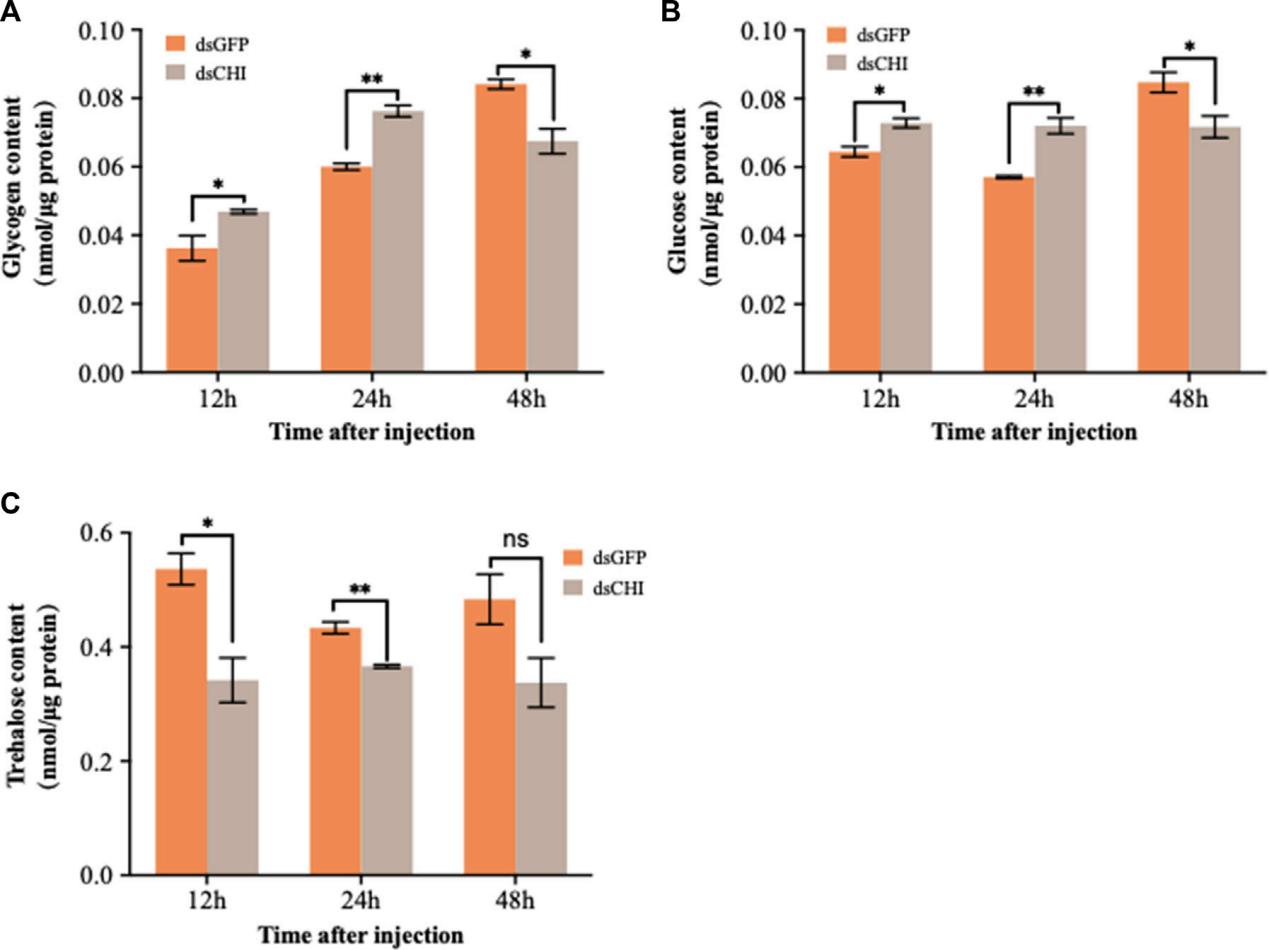
Changes in carbohydrates after RNAi. *Spodoptera frugiperda* larvae at the first day of the third instar stage were divided into three groups and injected with dsGFP and dsCHI, respectively. Insects were collected and used to determine glycogen content **(A)**, glucose content **(B)** and trehalose content **(C)** at 12, 24 and 48 h after dsRNA injection. Three replicates were performed per group. (Mean ± SE; t - test; **p < 0.01, *p < 0.05, ns *p* > 0.05).

After RNAi of *CHI* gene, the contents of glucose and glycogen showed a consistent trend, that is, the contents of glucose and glycogen increased significantly and extremely significantly at 12 and 24 h after dsRNA injection, but decreased significantly at 48 h (Figures 4A,B). Trehalose content decreased, especially at 12 and 24 h (Figure 4C). These results indicate that the RNAi of *CHI* gene of *S. frugiperda* will indirectly affect the supply of glucose and trehalose in the upstream of chitin synthesis pathway.

### Analysis of trehalase activity and expression after RNAi of ***CHI*** gene of *S. frugiperda*

**FIGURE 5:**
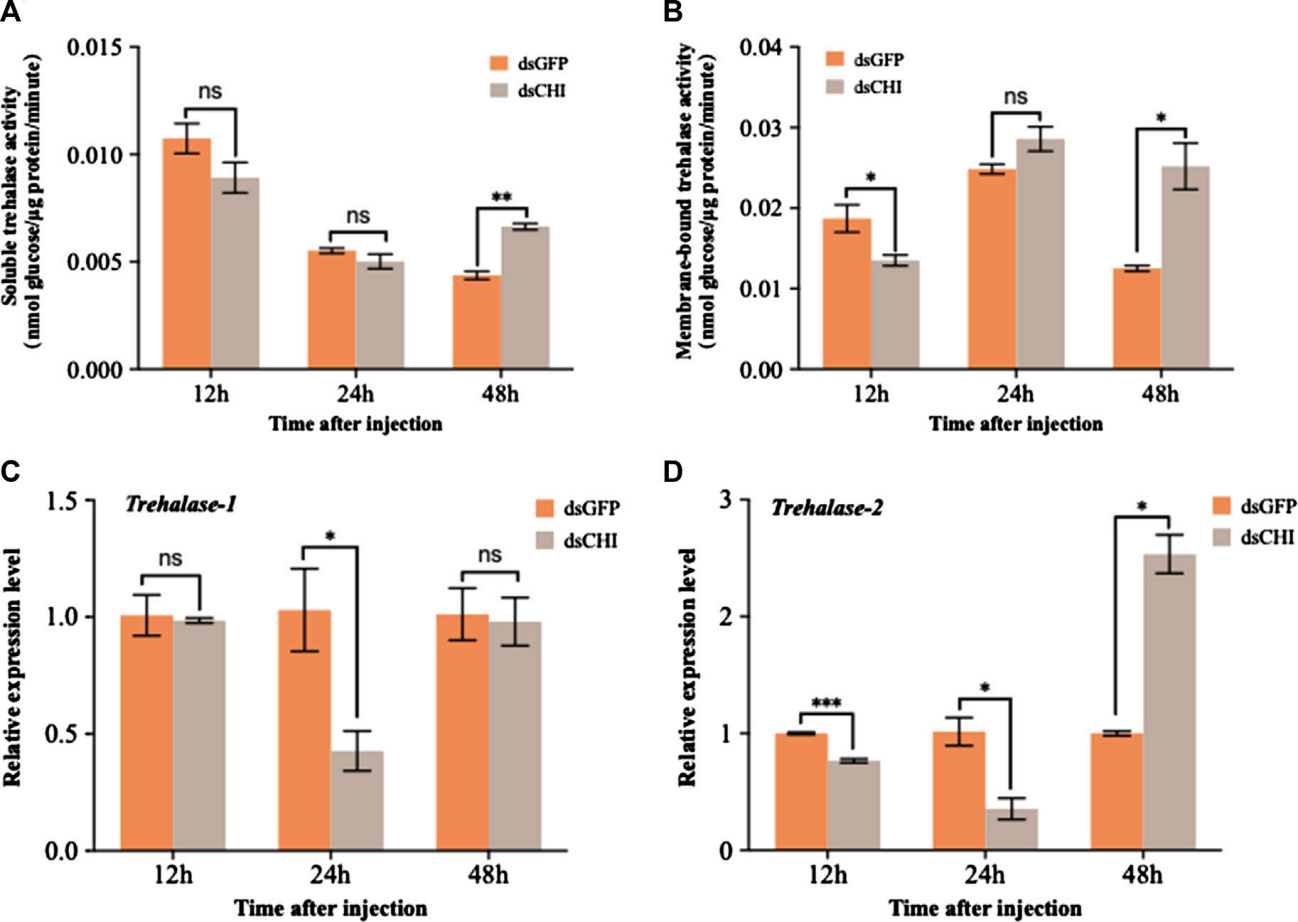
Changes in the activities of two trehalases and trehalase expression after RNAi. *Spodoptera frugiperda* larvae at the first day of the third instar stage were divided into three groups and injected with dsGFP and dsCHI, respectively. Insects were collected and used to determine soluble trehalase activity **(A)**, membrane-bound trehalase activity **(B)**, the relative expression level of trehalase-1 gene **(C)** and the relative expression level of trehalase-2 gene **(D)** at 12, 24 and 48 h after dsRNA injection. Three replicates were performed per group. (Mean ± SE; t - test; ***p < 0.001, **p < 0.01, *p < 0.05, ns *p* > 0.05).

The detection results of soluble and membrane-bound trehalase activities showed that the activities of these two kinds of trehalase enzymes increased significantly or extremely significantly at 48 h after RNAi of *CHI* gene of *S. frugiperda* (Figures 5A,B). At the same time, the expression of two trehalase genes was not consistent with the corresponding trehalase activity. Specifically, the expression of soluble trehalase gene (Trehalase-1) decreased significantly at 24 h (Figure 5C), while the trehalase activity did not change significantly at this time (Figure 5A); Compared with the control group, the expression of *trehalase-1* gene did not change at 48 h (Figure 5C), but the activity of soluble trehalase was significantly up-regulated (Figure 5A), which indicated that there may be other soluble trehalase genes in *S. frugiperda*, which affect the activity of soluble trehalase. This is also consistent with previous reports that insects have multiple soluble trehalase genes. In contrast, the membrane-bound trehalase activity and gene expression were relatively consistent. Compared with the dsGFP injection group, both significantly decreased at 12 h and significantly increased at 48 h (Figures 5B,D). According to the results in Figure 4C, trehalose decreased at 48 h after injection of dsCHI. It is speculated that the reason is that the activity of two types of trehalase enzymes increased, and then a large amount of trehalose was degraded. However, the contents of glucose and glycogen also decreased, so it is speculated that they may participate in other metabolism to provide energy, which requires further research.

### Expression of *TPS* and chitin synthesis pathway genes after RNAi of ***CHI*** gene of *S. frugiperda*

**FIGURE 6:**
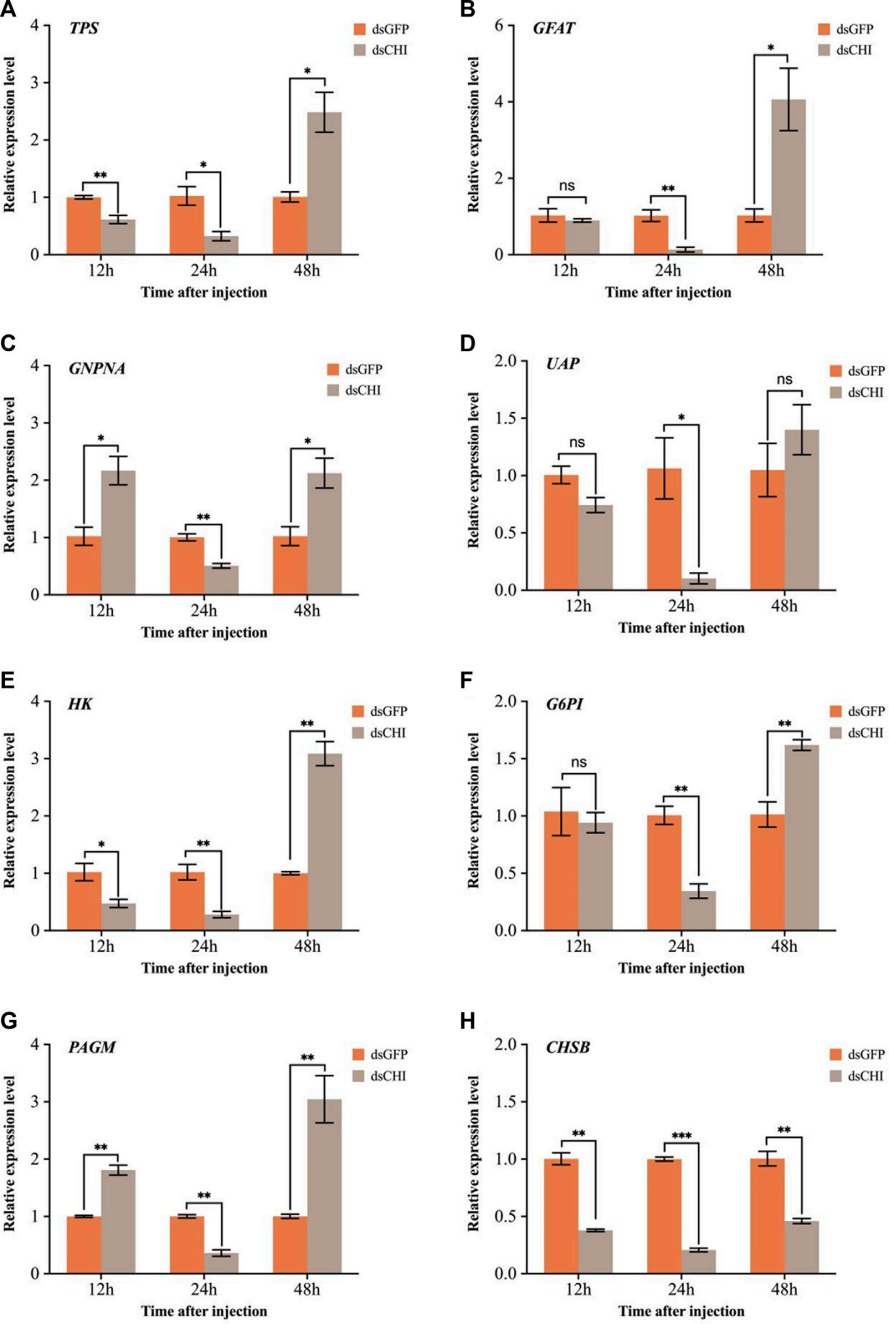
Changes in expression levels of genes related to trehalose and chitin metabolic pathways after RNAi. **(A)** *TPS,* trehalose-6-phosphate synthase; **(B)** *GFAT*, glutamine: fructose-6-phosphate amidotransferase; **(C)** *GNPNA*, glucosamine-6-phosphate-N-acetyltransferase; **(D)** *UAP*, UDP-N-acetylglucosamine pyrophosphorylases; **(E)** *HK*, hexokinase; **(F)** *G6PI*, glucose-6-phosphate isomerase; **(G)** *PAGM*, phosphoacetylglucosamine mutase; **(H)** *CHSB*, chitin synthase. *Spodoptera frugiperda* larvae at the first day of the third instar stage were divided into three groups and injected with dsGFP and dsCHI, respectively. Insects were collected and used to determine the relative expression levels of genes related to trehalose and chitin metabolic pathways at 12, 24 and 48 h after dsRNA injection. Three replicates were performed per group. (Mean ± SE; t - test; ***p < 0.001, **p < 0.01, *p < 0.05, ns *p* > 0.05).

The effects of RNAi of *CHI* gene on chitin synthesis pathway and trehalose-6-phosphate synthase (TPS) gene were further examined. The results showed that compared with the control group injected with dsGFP, the expression of trehalose synthase gene decreased significantly or extremely significantly at 12 and 24 h after injection of dsRNA of *CHI* gene (Figure 6A). Considering that trehalose enzyme activity and gene expression were not up-regulated (Figure 5), this should be the main reason for the decrease of trehalose content. At 48 h, the expression loss of trehalose synthase gene increased significantly (Figure 6A), but the trehalose content decreased. Based on the analysis of trehalose gene expression and enzyme activity, it should be that trehalose plays a major role in this stage. Although trehalose was synthesized, it was degraded more, resulting in a significant decrease in its content. In addition, the results showed that after *CHI* gene expression was inhibited, the expression trend of each gene in the chitin synthesis pathway of *S. frugiperda* was different. In addition to the significant decrease of key chitin synthase B (*CHSB*) from 12 to 48 h (Figure 6H), other genes including glutamine: fructose-6-phosphate amidotransferase (*GFAT*), glucosamine-6-phosphate-N-acetyltransferase (*GNPNA*), UDP-N-acetylglucosamine pyrophosphorylases (*UAP*), hexokinase (*HK*), glucose-6-phosphate isomerase (*G6PI*) and phosphoacetylglucosamine mutase (*PAGM*) all decreased significantly at 24 h, and then increased or significantly increased at 48 h (Figures 6B–G). These results indicate that the inhibition of *CHI* gene expression of *S. frugiperda* will also indirectly affect the upstream chitin synthesis pathway.

## Discussion

The *CHI* of *S. frugiperda* was first cloned in 2005. At that time, it was found that when the *CHI* was expressed, the content of chitin in the midgut peritrophic membrane could not be detected, indicating that this *CHI* could act on the degradation of chitin in the midgut (Bolognesi et al., 2005). The expression of insect chitinase gene is specific, and it is generally expressed in the midgut and integument (Ren et al., 2014). For example, *Cht5* and *Cht10* genes were almost not expressed in the midgut peritrophic membrane tissue of *Tribolium castaneum* and *Anopheles gambiae*, but they were highly expressed in the epidermis and body wall tissue (Zhu et al., 2008b; Zhang et al., 2011c). Reverse transcription-PCR detection showed that *CHI* was not only expressed in the midgut of wandering and pupal stages, It was also highly expressed in the epidermis (Bolognesi et al., 2005), indicating that there are tissue-specific chitinases in the gut and epidermis, respectively (Zheng et al., 2002). In addition, the expression of chitinase in different developmental stages is also specific. The study showed that *Cht2* was highly expressed in the second instar nymphs of *Phenacoccus solenopsis*, *Cht10*, *Cht3-3* and *IDGF* were highly expressed in female adults, while *Cht4* and *Cht4-1* were highly expressed in male pupae and male adults (Omar et al., 2019). The expression of *Cht7* gene in insects such as *Sogatella furcifera* and *Mythimna separata* changes significantly before and after molting (Chen et al., 2017; Yang and Fan, 2018), which is consistent with the results of our qRT-PCR detection. Similarly, we found that *CHI* was highly expressed in the late instar of *S. frugiperda* larvae and male adults (Figure 1), which indicate that it may also play a role in epidermal molting.

In the previous studies on the function of chitinase genes of *S. frugiperda* and *M. separata*, the bacteria mediated RNAi technology was used to inhibit the expression of *CHI* gene, and the larval body weight decreased or the wings of the adult were deformed after emergence (Ganbaatar et al., 2017; Wan et al., 2021). In this study, after dsRNA injection of *CHI* gene, although the expression of this gene increased at 48 h, the chitinase activity decreased (Figure 2). Other unidentified chitinase genes of *S. frugiperda* predicted were searched in the National Center for Biotechnology Information Search database, so it was speculated that the decline of chitinase activity may be caused by the joint action of multiple gene sequences, or there may be a time lag between changes in gene expression and enzyme activity. In addition, *S. frugiperda* also had difficulty in larval molting and could not normally develop to the old larvae (Figure 3B), indicating that after *CHI* expression was inhibited, chitin synthesis and degradation were affected, and the chitin in the old epidermis and the chitin in the new epidermis could not be degraded in time. These results showed that the *CHI* gene could affect epidermal molting, which was consistent with the study of chitinase function of other insects, Including *Drosophila* (Pesch et al., 2016; Dong et al., 2020), *T. castaneum* (Zhu et al., 2008b; Noh et al., 2018), *Nilaparvata lugens* (Xi et al., 2015), *Chilo suppressalis* (Zhao et al., 2018), *P. solenopsis* (Omar et al., 2019), *A. gambiae* (Zhang et al., 2011b), *Ostrinia furnacalis* (Qu et al., 2021), *S. furcifera* (Yang et al., 2021) *Plutella xylostella* (Zhu et al., 2019) and *Locusta migratoria* (Li et al., 2011: Li et al., 2015; Zhang et al., 2018). In addition, after *CHI* expression was inhibited, the epidermis of some larvae developed normally, but the contents of the body directly overflowed during dissection, which indirectly indicated that the structure of the midgut peritrophic membrane might be affected. This is consistent with the previous report that chitin content could not be detected in the midgut peritrophic membrane during *CHI* expression of *S. frugiperda* (Bolognesi et al., 2005). It is speculated that this *CHI* can also directly degrade chitin in the midgut peritrophic membrane.

As the blood sugar of insects, trehalose can be synthesized by trehalose-6-phosphate synthase (TPS)/trehalose-6-phosphate phosphatase (TPP) pathway (Tang et al., 2014; Tang et al., 2018a). It not only provides energy during insect development, but also degrades into glucose through trehalose, thus playing a very important role in insect chitin synthesis (Zhu et al., 2016; Tang et al., 2018b), regulating the synthesis and molting process of insect chitin (Zhang et al., 2019). In this study, it was found that after the *CHI* gene expression of *S. frugiperda* was inhibited, glucose and glycogen showed a trend of rising first and then decreasing, while trehalose showed a significant decline or decreasing trend (Figure 4), indicating that the change of chitin degrading enzyme expression would also affect or feed back to the change of trehalose expression and activity in the upstream of chitin synthesis pathway (Figure 5), but few studies related to this were reported. This is different from our previous findings on the positive regulation of chitin metabolism. It was previously found in *N. lugens* that the expression of trehalose metabolic genes was inhibited, which affected the changes of trehalose and glucose content. For example, after the trehalose gene expression was inhibited, the trehalose content basically decreased and the chitin synthase was down regulated (Zhao et al., 2016). When the expression of different trehalose genes was inhibited, the expression of chitinase genes was different (Zhang et al., 2017), but after the trehalose inhibitor treatment, both chitin synthase and *Cht1-Cht9* expression decreased significantly, resulting in a decrease in chitin content, and only the expression of *Cht10*, *IDGF* and *ENGase* genes was up-regulated (Tang et al., 2017). In this study, when the *CHI* expression of *S. frugiperda* decreased, the trehalose content also decreased (Figure 4C), affecting the expression of *TPS* (Figure 6A) or increasing the trehalase activity (Figure 5). These results are consistent with the inhibition of trehalase gene expression, indicating that there is a large correlation between trehalose and chitin metabolism, and any changes in gene expression or enzyme activity, can affect the energy supply and chitin synthesis and other molting processes.

In the study of *N. lugens TPS* gene, it was found that after the functional expression of *TPS1* and *TPS2* was inhibited, almost all chitinase genes showed a downward or downward trend, and chitin synthase showed a downward trend first and then an upward trend (Yang et al., 2017). However, after *TPS3* expression was inhibited, the expression of chitinase and various genes in the chitin synthesis pathway were different, but the chitin content was significantly decreased after the inhibition of the three *TPSs* (Zhou et al., 2022), which was consistent with the significant decrease of chitin content after the inhibition of *TPS* and trehalase expression in the *Acyrthosiphon pisum* (Wang et al., 2021), In contrast to the increase of chitin content after the inhibition of chitinase gene expression of *S. frugiperda* (Figure 3C). In this study, when *CHI* gene expression of *S. frugiperda* was inhibited, chitinase activity decreased (Figure 2B) and *CHSB* (Figure 6) expression decreased, indicating that *CHI* in *S. frugiperda* was related to midgut chitin synthesis. After all, *CHSB* was mainly responsible for the synthesis of midgut peritrophic membrane chitin (Arakane et al., 2005). Therefore, after the gene expression of trehalose metabolism and chitin metabolism is inhibited, although both can lead to chitin synthesis or degradation disorder in insects, the decrease in chitinase expression should be caused by the failure of chitin in the old epidermis to be degraded in time, resulting in an increase in chitin content (Figure 3C), while the inhibition of chitin synthase expression leads to a decrease in chitin content (Arakane et al., 2004). As for the two key genes in the trehalose metabolism pathway, although there are functional differences between several genes in the trehalase and trehalose-6-phosphate synthase families, the more important is that they act on the chitin synthase and affect the synthesis of chitin. The old epidermis can be degraded normally, but the new expression fails to form normally, resulting in the reduction of chitin content, which ultimately regulates molting and development (Tang et al., 2018b). These results indicate that the decrease and increase of chitin content, or the occurrence of any obstacle in the chitin synthesis and degradation system, can cause the chitin metabolism process of insects and lead to molting and development difficulties (Li et al., 2022). Although the regulation mechanism may be different, it also gives scientists the idea to find suitable target genes from them and develop potential chitinase, chitin synthase, trehalase and trehalose-6-phosphate synthase inhibitors are used for pest control (Zhang J. Y. et al., 2021; Wang et al., 2022).

Chitin is the main component of insect epidermis and peritrophic membrane, and is absent in plants and mammals. Therefore, the important genes of chitin synthesis pathway can be used as important potential pesticide targets, and have important research value and application potential. It was found that treatment of insect larvae with chitinase inhibitors can lead to dysmorphism such as molting and death (Liu et al., 2017), which indicates that insect development can be controlled by insect chitinase to control pests. Developing chitinase inhibitors is a potential biological insecticidal pathway and method (Spindler et al., 1990; Chen and Yang, 2020; Chen et al., 2021; Li et al., 2022). However, how the changes of chitinase gene and enzyme activity expression cause the upstream trehalose metabolism pathway. Experiments need to be carried out to explore whether trehalose metabolism related genes are regulated by chitin synthesis pathway, other transcription factors or MicroRNA. The outbreak and harm of pests have a serious impact on grain production, especially the migratory pests such as *S. frugiperda*, whose occurrence activities will seriously reduce the production of corn and other crops. Therefore, monitoring and controlling pests has become a key research topic. Some studies have reported that the insecticidal effect of chitinase inhibitor alone is not particularly obvious (Liu et al., 2019). If similar compounds can be found to act on trehalose and chitin metabolic pathways at the same time, they will play a greater role in the future control of agricultural and forestry pests.

## Data Availability

The datasets presented in this study can be found in online repositories. The names of the repository/repositories and accession number(s) can be found in the article/Supplementary Material.
