# Supplementary material for: Chitinase (CHI) of Spodoptera frugiperda affects molting development by regulating the metabolism of chitin and trehalose
Source: Front Physiol. 2022 Oct 3;13:1034926. doi: 10.3389/fphys.2022.1034926 (PMC9574123; doi:10.3389/fphys.2022.1034926)
Supplement: Supplementary file 1 [file Table1.DOCX]

Table S1 Primer sequences for dsRNA synthesis and Real time fluorescence quantitative PCR

| Gene name | GenBank ID | Forward primer (5’-3’) | Reverse primer (5’-3’) | Length of product | Application |
| --- | --- | --- | --- | --- | --- |
| *CHI* | XM_035597059.1 | ATCGGTTCCTCTTCCTCGTC | GAGGGTTCTCGTCTCCACAA | 678 bp | PCR |
| *GFP* | MN728541.1 | AAGGGCGAGGAGCTGTTCACCG | CAGCAGGACCATGTGATCGCGC | 657 bp |  |
| *CHI* | XM_035597059.1 | ATTGACCCTGAGTTGGACGT | GCCATTCGCGTTTGTTTCTG | 158 bp | qRT-PCR |
| *CHSB* | MZ364352 | GTAAGAAGCCGGACGACCTA | CAATCTCCTCTTGGCGTTGG | 212 bp |  |
| *Trehalase-1* | DQ447188.1 | TCAGATGAAGGTGAACTCGAAGA | GGAATGATGAATCCGTGGGTA | 209 bp |  |
| *Trehalase-2* | EU872435.1 | CTGCTGCTGTCGGAGATGA | TAGGAGGGGAGGCTGTGAT | 132 bp |  |
| *TPS* | MT920672.1 | TGGTCGGCTTCCACATAACT | AATCGGTCGAAGGGTACTCC | 157 bp |  |
| *HK* | XM_035594594.1 | AGGTGTTGGAAGAACTCGGT | TGAGGGTGGAAACGGTAGAC | 190 bp |  |
| *G6PI* | XM_035596038.1 | TGGATCAGCACTTCACCACT | CTCTCCATGTCGCCTTGTTG | 169 bp |  |
| *GFAT* | XM_035573563.1 | ACCAAGATTCGACAGGTGCT | TCCGGCCATAATACCCTCAC | 177 bp |  |
| *GNPNA* | XM_035593197.1 | ACAGAGGAAAGCAGTTGGGA | GCGTTAGAGTTTCCGGGTTC | 154 bp |  |
| *PAGM* | XM_035586036.1 | TCGATCAAGATTGACGTGAG | AGACCACATGTTCTAACAAGA | 263 bp |  |
| *UAP* | XM_035601390.1 | ACCTAAACTCTCACGGGCAA | TTCGGCACTGCTTCGTAATG | 211 bp |  |
| *RPL10* | OK319023.1 | GACTTGGGTAAGAAGAAG | GATGACATGGAATGGATG | 189 bp |  |
